# Bridging the Gap in Carbon Free Iron Making: How Hydrogen Affects the Reduction of Iron Ore between 900 and 1590 °C

**DOI:** 10.1021/acssuschemeng.5c03402

**Published:** 2025-10-16

**Authors:** Ram Krushna Mohanta, Dallin Fisher, Yuri Korobeinikov, Qijun Hong, Christopher Muhich, Seetharaman Sridhar, Noemi Leick

**Affiliations:** 1 7864 Arizona State University, Tempe, Arizona 85281, United States; 2 53405 National Renewable Energy Laboratory, Golden, Colorado 80401, United States

**Keywords:** Hydrogen reduction, Hematite ore reduction, Green steel, Solid−liquid slag phase

## Abstract

Hydrogen-based reduction of iron ore for iron and steel production has emerged as a promising alternative to coal and natural gas. Unlike other hydrogen-based iron ore reduction studies, this research focuses on a wide temperature range across 900–1590 °C, encompassing reduction in solid, mixed, and liquid (slag) phases. For a 20 min exposure to hydrogen, the reduction degree increased monotonically from ∼35% at 900 °C to >90% at 1550 °C, except between 1100 °C and 1400 °C, where it stagnated ∼60%. This experimental work challenges the widely accepted notion that higher temperatures enhance the reduction process. Instead, it reveals an overlooked kinetic bottleneck, suggesting complex thermodynamic and mass transfer limitations influenced by phase transformations, diffusion barriers, and microstructural changes. Density functional theory-based molecular dynamics simulations indicate that oxygen diffusivity in BCC iron is 3.88 × 10^–5^ cm^2^/s which is ∼5–10 times higher than that in FCC iron. This study reports that this stagnant reduction degree in the mixed solid–liquid phase is due to competition of multiple mechanisms, such as surface- and bulk-diffusion, pore collapse mechanisms, and crystallographic transitions.

## Graphical abstract

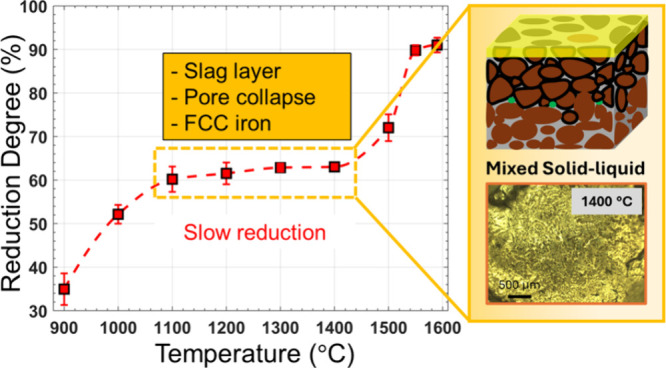

## 1 Introduction

Steel production is highly energy-intensive, making energy resiliency a critical consideration for future iron processing.^1,2^ In this context, hydrogen (H_2_)-based technologies have recently gained attention.^3–5^ Iron ore reduction using H_2_ appears particularly promising, as studies suggest it could be economically integrated with electric arc furnaces.^1,6^ Beyond its environmental benefits and decarbonization potential,^7–9^ since it primarily produces water (H_2_O) as a byproduct, H_2_ also offers advantages in energy efficiency and process scalability.^6,9^

Theoretical studies and the Richardson–Ellingham diagram suggest that H_2_ can effectively reduce iron ore,^10^ making it a viable alternative for steel manufacturing.^11^ Therefore, the role of H_2_ as a reducing agent has primarily been researched for solid-state and liquid-phase iron ore reduction regimes, covering temperatures of <1300 °C^12–26^ and >1400 °C,^27,28^ respectively. While some studies have explored H_2_ reduction kinetics at elevated temperature,^29,30^ key limitations persist, such as the use of powders, synthetic plates, or narrow temperature windows, often without in situ phase tracking. Earlier work by Shigematsu and Iwai^30^ demonstrated that small amounts of SiO_2_ and CaO accelerate wüstite reduction at <1100 °C; however, at >1200 °C the additives no longer accelerate the reduction process. Rather, dense iron layer formation and the low reducibility of silicate phases are hindering the reduction, highlighting the critical role of gangue chemistry.

This study addresses these gaps by investigating the H_2_-driven reduction kinetics of direct reduced iron (DRI)-grade hematite ore from 900 °C to 1590 °C, with a focus on the formation and behavior of solid–liquid mixed (slag) phases. Real-time reduction processes are monitored using confocal scanning laser microscopy (CSLM), enabling in situ visualization of evolving phase transformations complemented by postreduction characterization via scanning electron microscopy (SEM), further elucidating mechanisms active in intermediate temperature regimes. Additionally, density functional theory (DFT)-based molecular dynamics (MD) simulations are employed to analyze oxygen diffusion in body-centered cubic (BCC) and face-centered cubic (FCC) iron, offering atomic-level insight into reduction dynamics.

## 2 Results and Discussion

The weight change and oxygen content of the reduced samples were measured as outlined in the experimental methods (Supporting Information, including Figure S1). From these values, the reduction degree, metallization percentage, and sample weight as a function of temperature, ranging from 900 °C to 1590 °C, and over a 20 min reduction interval were extracted and graphed in Figure 1a. The reduction degree quantifies the amount of oxygen removed from the sample, while the metallization percentage indicates the proportion of metallic iron formed after the reduction. From the trends in Figure 1a, we identified three key reduction regimes, 900–1100 °C, 1100–1400 °C, and 1400–1590 °C, associated with a solid phase, mixed solid–liquid phase, and liquid phase reduction, respectively. These phases were deduced from the in situ imaging taken by the CSLM for each reduction temperature at the 20 min mark, shown in Figure 1b–d and Figure S2. While the melting point of Fe lies at 1538 °C, iron oxides and slag mix become significant to the liquid phase at ∼1400 °C, as shown in Figure 2a.

**Figure 1:**
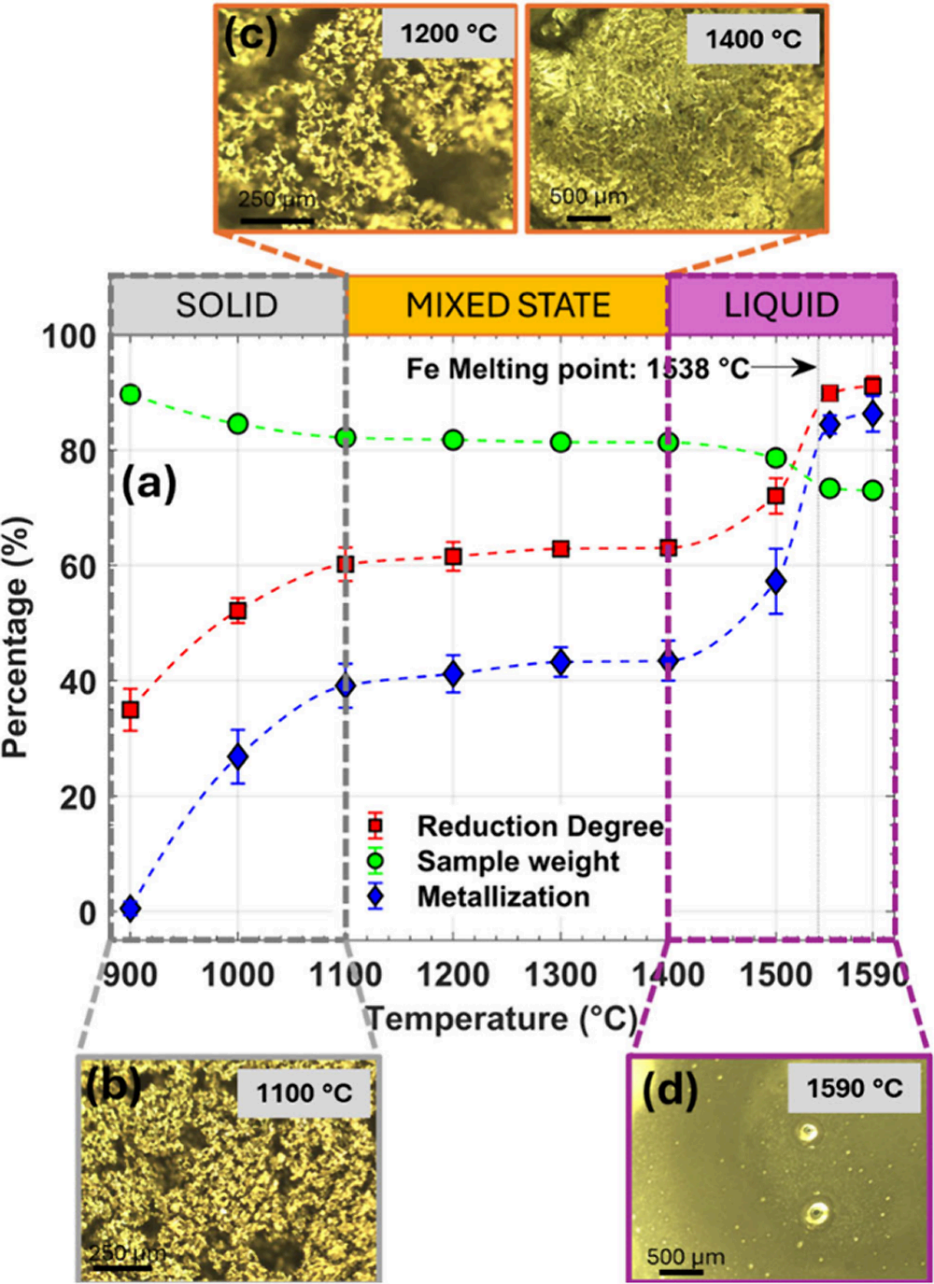
(a) Variation of reduction degree, sample weight, and metallization degree measured in %, after exposure to 5% H_2_ diluted in argon at different temperatures where each sample was reduced for 20 min, and the dotted lines are guides to the eye. The insets (b)–(d) correspond to CSLM images taken at the temperature indicated.

**Figure 2:**
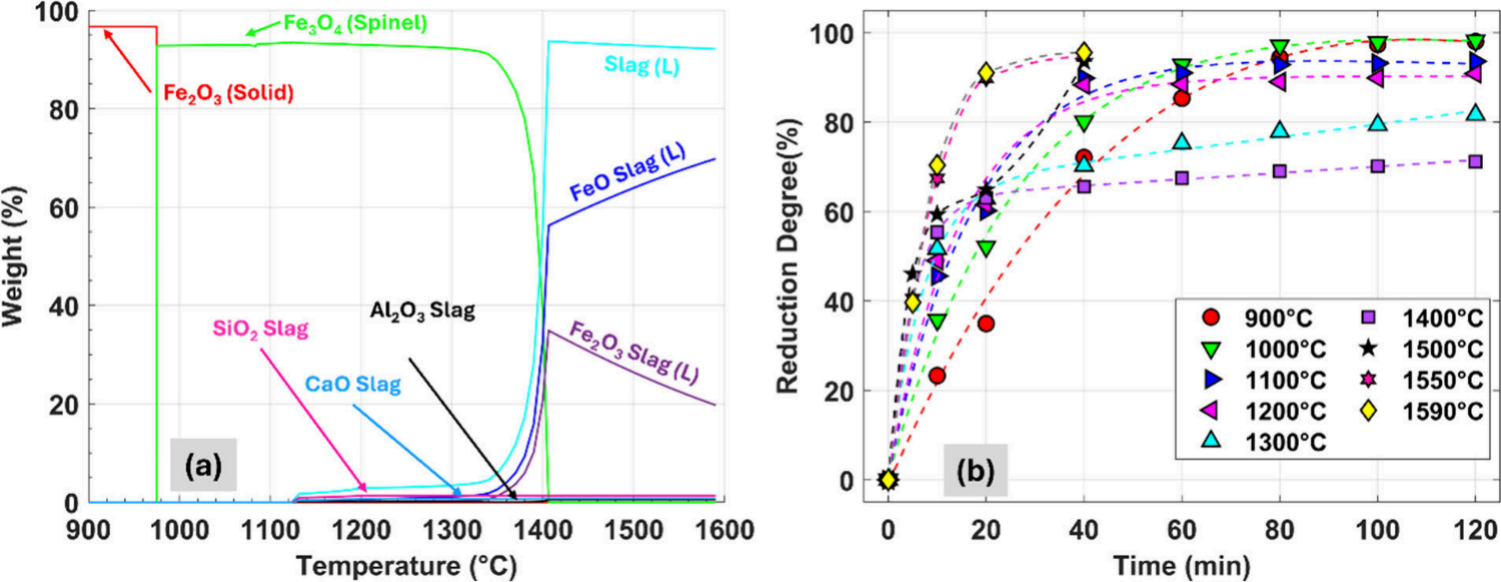
(a) Equilibrium phases of the DRI hematite ore during heating cycle in argon environment as calculated by FactSage 8.2 (database: FactPS, FToxide). S: Solid. L: Liquid. (b) Variation of reduction degree with respect to reduction interval for different temperatures between 900 °C and 1400 °C. For equipment safety reasons, the reduction time was limited to 0–40 min for temperatures of >1400 °C.

Although it is widely reported in the literature^15,23,24,31,32^ that higher temperatures enhance reaction kinetics due to increased diffusion and accelerated phase-boundary reactions, the results from Figure 1 suggest this is only the case when the ore is either predominantly solid (900–1100 °C) or liquid (≥1400 °C) but not when the ore is a mixed solid–liquid state. In the solid phase regime, the reduction degree after 20 min H_2_ treatment increases sharply by 22% and by ∼30% between 1400 °C and 1590 °C, in the liquid phase. However, it is remarkable that between 1100 °C and 1400 °C, the reduction degree remains stable, with only a minor increase of ∼3%. Initially, this unusual behavior was attributed to the fact that 20 min was insufficient to complete the reduction process, as shown in Figure 2b, and was therefore an artifact of the dynamic reduction process. However, plotting the reduction degree as a function of temperature for reduction times of ≥40 min, depicted in Figure S3, shows that the reduction degree does not increase or remain stable in the mixed solid–liquid phase temperature range but rather decreases between 1100 °C and 1400 °C.

In the solid-state reduction regime, 900–1100 °C, the chemical reaction itself is rate-limiting, as reaction rates are inherently slower and more sensitive to temperature due to a larger activation energy compared to transport processes.^33,34^ However, the porous structure of hematite ore, shown in Figure 1b, facilitates gaseous H_2_ diffusion to the oxide interface; hence an increase in the reduction degree is observed between 900 and 1100 °C, consistent with previous reports.^16^ In this stage, the reduction process proceeds as follows: H_2_ molecules from the gas stream reach the laminar layer at the oxide interface, where they are absorbed. Oxygen is removed from iron oxide through a phase-boundary reaction, producing water as a gaseous byproduct, leaving either FeO or Fe as a solid byproduct. The FeO or metallic Fe nuclei then undergo growth via solid-state diffusion, while water vapor diffuses back to the laminar layer. Also, due to the reduction of Fe_2_O_3_ to FeO, new pore formation takes place as FeO has smaller grain structure compared to Fe_2_O_3_.^35^

The sharp increase in reduction degree at ≥1400 °C is primarily attributed to the elevated temperatures, creating thermodynamically favorable conditions for the reduction process. From the FactSage phase diagram shown in Figure 2a, at these temperatures the proportion of liquid slag within the sample rises significantly, reaching 94%, with FeO contributing ∼60%, Fe_2_O_3_ accounting for ∼37%, and gangue-related components forming the remaining ∼3%. The elevated FeO concentration in the liquid slag further reduces the melting temperature of the iron ore mixture (Figure S4),^36^ thereby promoting the formation of additional liquid phases. At ≥1500 °C, the sample transitions entirely into a liquid state with negligible solid iron oxide phases remaining, transforming the reduction into a purely gas-melt reaction. At these temperatures, metallization also rises sharply, reaching 86% at 1590 °C as shown in Figure S5, indicating near-complete reduction of FeO_*x*_ to metallic Fe. This steep increase in metallization is attributed to the accelerated reduction kinetics at high temperatures, where iron oxides approach their melting points, facilitating rapid oxide-to-metal conversion and easy phase separation. Based on the few reduction studies in the liquid phase,^27,28^ in this regime, oxygen diffusion to the slag surface is facilitated, creating a more efficient interaction with H_2_ and accelerating the reduction kinetics. Beyond the melting point of Fe (1538 °C), it precipitates as liquid droplets from the slag, as shown in Figure 1d, subsequently coalescing into larger clusters and inducing morphological changes in the melt.

Reduction reactions are strongly favored thermodynamically at these elevated temperatures (≥1400 °C), with a negative Gibbs free energy for FeO reduction to Fe, providing a significant driving force for the reaction. At the same time, the solubility of FeO in the slag increases with temperature,^37^ creating a more homogeneous distribution of FeO in the slag, thus more reaction interfaces. A higher FeO solubility also decreases the slag viscosity,^38,39^ promoting oxygen diffusion within the melt. Moreover, H_2_ solubility in molten oxides increases at higher temperatures,^40^ further enhancing the reduction process. While these thermochemical trends indicate favorable kinetics, the actual reduction mechanism is highly complex, likely governed by multiple temperature-dependent, phase-coupled rate-limiting processes. Preliminary kinetic assessment using a master plot analysis^41^ (see Supporting Information) indicates that rigorous extraction of apparent activation energy would require a separate dedicated study with finer time resolution and narrower temperature intervals.

Focusing on the mixed solid–liquid reduction regime, between 1100 and 1400 °C, it is hypothesized that there are at least three possible phenomena impacting mass transport of H_2_ to the oxide interface and oxygen diffusion and solubility through reduced metallic Fe back to the laminar layer: (a) slag formation, (b) collapse of the pore structure, and (c) crystallographic phase transitions. Formation of liquid slag, shown in Figure 2a, is initiated at ∼1130 °C and reaches ∼4.5% at 1340 °C. As soon as H_2_ accesses iron oxide and reduces Fe_2_O_3_ to FeO, the slag melting point quickly drops and the fraction of liquid rises,^42^ as shown in Figure S5. Further, reduction reactions are confined to the surface. As the surface is gradually reduced, a layer of solid Fe forms, changing the pore structure to a dense top layer, thereby hindering oxygen diffusion to the interface and slowing down the reduction process.

An example of a solid low-porosity metallic Fe is depicted in Figure 3, where the sample reduced to 1300 °C exhibits an unreduced core beneath the metallic surface. The metallic Fe concentration gradually decreases toward the core, a behavior linked to extensive slag formation infiltrating the porous structure and obstructing gas transport. Similar inhibition was reported by Shigematsu and co-workers,^30^ who found that while small additions of SiO_2_ and CaO accelerate FeO reduction at <1100 °C, at >1200 °C the process is hindered by dense Fe layers and the poor reducibility of silicate phases, such as fayalite. Their study employed thin, dense slabs with a homogeneous distribution of SiO_2_ and CaO, providing valuable insights. In this work, industrial DRI-grade pellets exhibit intrinsic porosity, which creates changing gas pathways and pore collapse. Meanwhile, nonuniform gangue distribution creates localized slag formation and infiltration. The in situ images in Figures S3 and S8 validate the mechanisms identified in the dense-slab system and extend them by directly visualizing slag penetration and pore collapse up to 1590 °C. Importantly, this intrinsic porosity allows quantification of pore collapse and sealing with dense product (metallic iron) layer as a key contributor to reduction inhibition in the mixed-phase regime.

**Figure 3:**
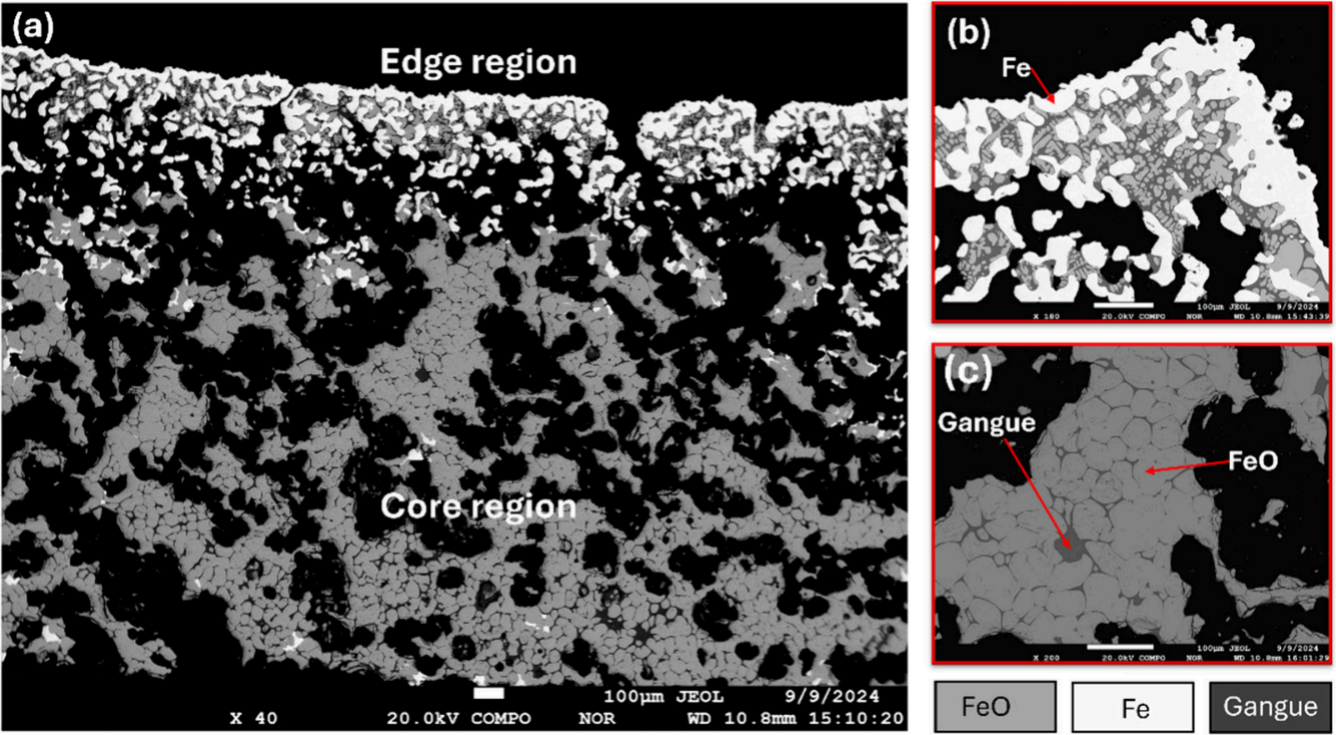
(a) Backscattering SEM image of sample reduced at 1300 °C depicting the solid Fe layer on top (in the edge region), and unreacted FeO-Fe_2_O_3_ underneath (in the core region). (b) Edge region. (c) Core region.

The porosity analysis of the reduced sample, graphed in Figure 4a, indicates a ∼55% decline in edge region porosity beyond 1100 °C, which in turn reduces the overall porosity of the sample by ∼25%. This structural collapse of pores in the edge region is likely to decrease the surface area for oxygen–hydrogen reactions to occur, thereby decelerating the reduction process. Additionally, core region porosity follows a similar trend, albeit remaining consistently lower than the edge region, suggesting a compact structure in the core that may further limit the removal of reaction byproducts back into the laminar layer, affecting the reduction process.

**Figure 4:**
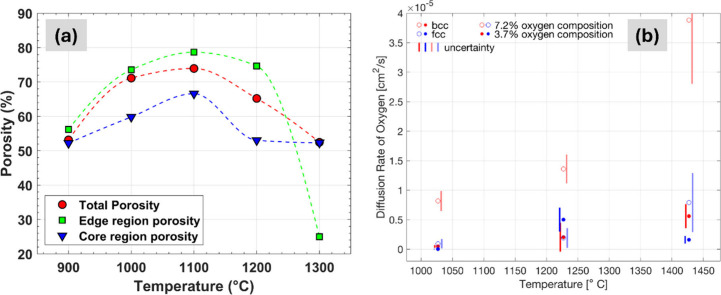
(a) Porosity of different sample regions at different temperatures after exposure to 5% H_2_ in argon for 20 min. The porosity values are calculated from the backscattered SEM images, exemplified in Figure 3. (b) Calculated diffusion coefficient of oxygen in BCC and FCC iron at 1027, 1227, and 1427 °C (1300, 1500, and 1700 K) for two oxygen concentrations, 3.7 and 7.2 atomic %. Uncertainties are determined based on four molecular dynamic simulations for each temperature, phase, and composition.

Considering the oxygen solubility and diffusion in this mixed-phase temperature regime, the impact of phase transitions between FCC and BCC Fe was computationally evaluated by using Brownian motion theory. DFT-MD simulations were employed to quantify the diffusion rate, with detailed methodologies provided in the Supporting Information. The DFT MD simulations were performed through a SLUSCHI package,^43^ which automates the calculation process of diffusion coefficient. The simulations aim to quantitatively confirm and extend the understanding of oxygen transport under the specific conditions relevant to our work, i.e., over broad temperatures and non-negligible oxygen concentrations. Figure S6 shows the mean displacement of each species over time, and linear regression was applied to determine the diffusion coefficients using eq 1,

1 $$r^{2}=6Dt$$

where *r* is distance, *t* is time, and *D* is the diffusion coefficient, graphed in Figure 4b for three temperatures. Table 1 summarizes the diffusion coefficients and demonstrates that oxygen atoms (O) diffuse 5–10× faster in BCC iron within the temperature range 1027–1427 °C (1300–1700 K). Our calculated diffusion coefficient of O in BCC iron (8.2 × 10^–10^ m^2^/s at 1023 °C) aligns closely with experimental reports (4.3 × 10^–10^ m^2^/s at 1068 °C).^44^ Additionally, considering Fick’s first law, *J* = *D*·Δ*c*/*L*, where *J* is the mass/molar flux, *D* the diffusion coefficient, and Δ*c*/*L* the oxygen concentration gradient over the iron layer thickness *L*, these findings corroborate that the phase transition from BCC to FCC iron, with FCC iron being stable from 912 to 1394 °C and low oxygen solubility in iron (see Figure S12), substantially slows down oxygen diffusion through the Fe layer and consequently slows down the reduction process.

**Table 1:** Overview of the Computed Oxygen Diffusion Coefficients (*D*) in BCC and FCC Iron at 1027, 1227, and 1427 °C^a^

temperature (°C)	*D*_O_ in BCC (10^–6^ cm^2^/s)	*D*_O_ in FCC (10^–6^ cm^2^/s)
1027	8.2	0.9
1227	13.6	2.2
1427	38.8	7.9

a The simulation shows that the diffusion coefficients are significantly higher in BCC iron.

## 3 Conclusion

The H_2_-driven reduction process of DRI grade hematite ore across three temperature regimes is schematically summarized in Figure 5, highlighting the dominant mechanisms at each stage. In the temperature range of 900–1100 °C, the reduction occurs in the solid state, where H_2_ diffuses through the porous oxide layer, and is governed by surface-controlled kinetics. The reduction degree increases from approximately 35% to 60% over 20 min, accompanied by a ∼30% rise in porosity, which facilitates gas diffusion. However, in the intermediate temperature range of 1100–1400 °C, the reduction degree stagnates around 60% despite rising temperature, indicating a kinetic bottleneck. This is attributed to the formation of a liquid slag layer and pore structure collapse, which collectively reduce the porosity by ∼25% and restrict gas transport to and from the core. Additionally, Fe formation in the FCC phase, stable from 912 to 1394 °C, exhibits lower oxygen diffusivity than its BCC counterpart, as supported by DFT simulations, further hindering the reduction once a solid Fe layer appears and impacts oxygen solubility and diffusion.

**Figure 5:**
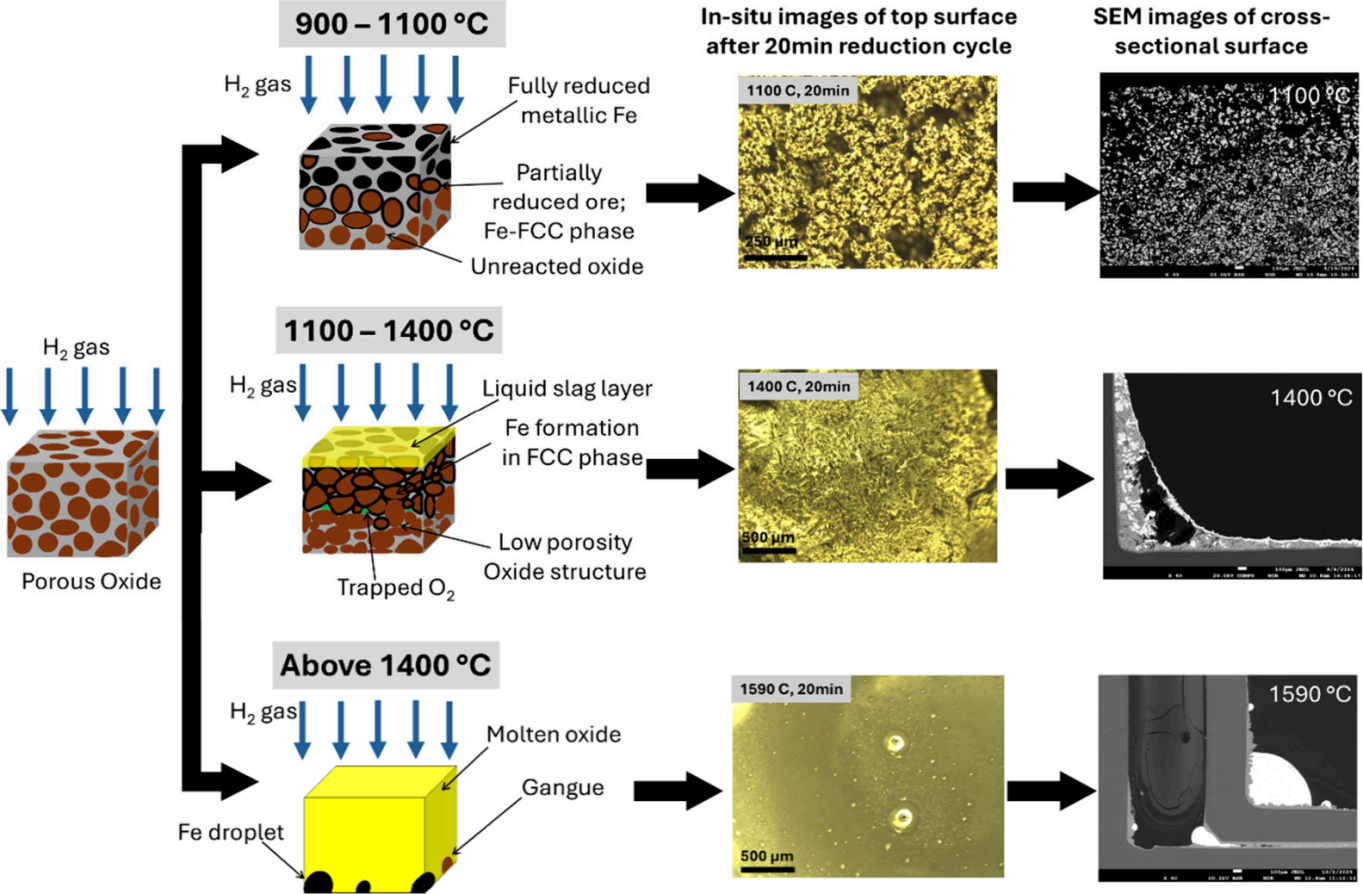
Schematic overview of the hydrogen-driven reduction process in the three regimes observed in this study: solid-state reduction between 900 °C and 1100 °C, mixed solid–liquid phase between 1100 °C and 1400 °C where the reduction process is observed to slow down significantly, and finally the liquid phase reduction between 1400 °C and 1590 °C.

Above 1400 °C, the reaction shifts to a gas-melt phase, where the high temperature provides favorable conditions for rapid reduction, allowing metallic Fe to separate efficiently from the molten oxide. Further, the high temperature lowers the Gibbs free energy of FeO reduction and increases the FeO solubility in the slag such that ∼85% metallization is reached at 1590 °C after 20 min. The intermediate temperature range of 1100–1400 °C therefore presents significant challenges due to the combined effects of mass transport limitations and oxygen diffusion constraints, negatively impacting the overall reduction efficiency.

One of the key findings of the study is the dependence of the reduction and metallization degrees on the temperature when using H_2_ as the reducing agent of iron ore. In contrast to previous reports stating that the reduction degree monotonically increases with temperature, the current observation suggests that the reduction process in fact slows down between 1100 °C and 1400 °C due to the formation of a mixed solid–liquid phase. At least three phenomena are proposed to explain this behavior: liquid slag formation, pore structure collapse, and crystallographic considerations. The hypothesized mechanisms each impact mass transport of gas-phase species, suggesting potential thermodynamic and kinetic correlations among the observed mechanisms, which merit further exploration.

This study offers a systematic characterization of the reduction behavior of hematite ore across a wide temperature range, providing critical insights into optimizing reduction conditions for H_2_-based ironmaking.

## Supplementary Material



## Notes

Experimental details and computational methods, in situ images obtained using CSLM for all temperatures considered in this manuscript, reduction degree and metallization degree, and molecular dynamics data relevant to this manuscript (PDF)

## References

[1] Azimi A., van der Spek M. (2025). Prospective
Life Cycle Assessment Suggests Direct Reduced
Iron Is the Most Sustainable Pathway to Net-Zero Steelmaking. Ind. Eng. Chem. Res..

[2] Jordan K. H., Jaramillo P., Karplus V. J., Adams P. J., Muller N. Z. (2025). The Role
of Hydrogen in Decarbonizing U.S. Iron and Steel Production. Environ. Sci. Technol..

[3] Zhu Y., Keoleian G. A., Cooper D. R. (2025). The Role
of Hydrogen in Decarbonizing
U.S. Industry: A Review. Renewable and Sustainable
Energy Reviews.

[4] Fei Y., Guan X., Kuang S., Yu A., Yang N. (2024). A Review on
the Modeling and Simulation of Shaft Furnace Hydrogen Metallurgy:
A Chemical Engineering Perspective. ACS Engineering
Au.

[5] Sabat K. C., Rajput P., Paramguru R. K., Bhoi B., Mishra B. K. (2014). Reduction
of Oxide Minerals by Hydrogen Plasma: An Overview. Plasma Chemistry and Plasma Processing.

[6] Rosner F., Papadias D., Brooks K., Yoro K., Ahluwalia R., Autrey T., Breunig H. (2023). Green Steel:
Design and Cost Analysis
of Hydrogen-Based Direct Iron Reduction. Energy
Environ. Sci..

[7] van
der Spek M., Banet C., Bauer C., Gabrielli P., Goldthorpe W., Mazzotti M., Munkejord S. T., Røkke N. A., Shah N., Sunny N., Sutter D., Trusler J. M., Gazzani M. (2022). Perspective on the Hydrogen Economy
as a Pathway to Reach Net-Zero CO2 Emissions in Europe. Energy Environ. Sci..

[8] Flores-Granobles M., Saeys M. (2020). Minimizing CO2emissions
with Renewable Energy: A Comparative Study
of Emerging Technologies in the Steel Industry. Energy Environ. Sci..

[9] Baldauf-Sommerbauer G., Lux S., Siebenhofer M. (2016). Sustainable
Iron Production from Mineral Iron Carbonate
and Hydrogen. Green Chem..

[10] Ellingham H. J. (1944). Transactions
and Communications. Journal of the Society of
Chemical Industry.

[11] D’Angelo A., Salucci E., Russo V., Grénman H., Saxén H. (2025). Hydrogen Reduction of Iron Oxide Powder in Thin Layers. Ind. Eng. Chem. Res..

[12] von Bogdandy, L. ; Engell, H.-J. The Reduction of Iron Ores; Springer Berlin Heidelberg, 1971; 10.1007/978-3-662-10400-2.

[13] Turkdogan E. T., Vinters J. V. (1971). Gaseous Reduction of Iron Oxides:
Part I. Reduction
of Hematite in Hydrogen. Metallurgical Transactions
1971 2:11.

[14] Turkdogan E. T., Olsson R. G., Vinters J. V. (1971). Gaseous Reduction of Iron Oxides:
Part II. Pore Characteristics of Iron Reduced from Hematite in Hydrogen. Metallurgical Transactions 1971 2:11.

[15] Spreitzer D., Schenk J. (2019). Reduction of Iron Oxides
with Hydrogen—A Review. Steel Res. Int..

[16] Spreitzer D., Schenk J. (2019). Iron Ore Reduction
by Hydrogen Using a Laboratory Scale
Fluidized Bed Reactor: Kinetic Investigation—Experimental Setup
and Method for Determination. Metallurgical
and Materials Transactions B: Process Metallurgy and Materials Processing
Science.

[17] Hou B., Zhang H., Li H., Zhu Q. (2012). Study on Kinetics of
Iron Oxide Reduction by Hydrogen. Chin J. Chem.
Eng..

[18] Wagner, D. ; Devisme, O. ; Patisson, F. ; Ablitzer, D. ; Ablitzer, D. A. A Laboratory Study of the Reduction of Iron Oxides by Hydrogen. 2008. https://hal.science/hal-00265636.

[19] Teplov O. A. (2012). Kinetics
of the Low-Temperature Hydrogen Reduction of Magnetite Concentrates. Russian Metallurgy (Metally).

[20] Fruehan R. J., Li Y., Brabie L., Kim E. J. (2005). Final Stage of Reduction of Iron
Ores by Hydrogen. Scandinavian Journal of Metallurgy.

[21] Ma Y., Souza Filho I. R., Zhang X., Nandy S., Barriobero-Vila P., Requena G., Vogel D., Rohwerder M., Ponge D., Springer H., Raabe D. (2022). Hydrogen-Based Direct
Reduction of Iron Oxide at 700°C: Heterogeneity at Pellet and
Microstructure Scales. International Journal
of Minerals, Metallurgy and Materials.

[22] Kim S. H., Zhang X., Ma Y., Souza Filho I. R., Schweinar K., Angenendt K., Vogel D., Stephenson L. T., El-Zoka A. A., Mianroodi J. R., Rohwerder M., Gault B., Raabe D. (2021). Influence of Microstructure
and Atomic-Scale
Chemistry on the Direct Reduction of Iron Ore with Hydrogen at 700°C. Acta Mater..

[23] Scharm C., Küster F., Laabs M., Huang Q., Volkova O., Reinmöller M., Guhl S., Meyer B. (2022). Direct Reduction of
Iron Ore Pellets by H2 and CO: In-Situ Investigation of the Structural
Transformation and Reduction Progression Caused by Atmosphere and
Temperature. Miner. Eng..

[24] Bahgat M., Khedr M. H. (2007). Reduction Kinetics, Magnetic Behavior
and Morphological
Changes during Reduction of Magnetite Single Crystal. Materials Science and Engineering: B.

[25] Perrone A., Cavaliere P., Sadeghi B., Dijon L., Laska A. (2025). Optimization
of Hydrogen Utilization and Process Efficiency in the Direct Reduction
of Iron Oxide Pellets: A Comprehensive Analysis of Processing Parameters
and Pellet Composition. Steel Res. Int..

[26] Korobeinikov Y., Meshram A., Harris C., Kovtun O., Govro J., O’Malley R. J., Volkova O., Sridhar S. (2023). Reduction of Iron-Ore
Pellets Using Different Gas Mixtures and Temperatures. Steel Res. Int..

[27] Ban-ya S., Iguchi Y., Nagasaka T. (1984). Rate of Reduction of Liquid Wustite
with Hydrogen. Tetsu-to-Hagane.

[28] Nagasaka T., Hino M., Ban-Ya S. (2000). Interfacial Kinetics of Hydrogen
with Liquid Slag Containing Iron Oxide. Metall.
Mater. Trans. B.

[29] Choi M. E., Sohn H. Y. (2010). Development of Green Suspension Ironmaking
Technology
Based on Hydrogen Reduction of Iron Oxide Concentrate: Rate Measurements. Ironmaking and Steelmaking.

[30] Shigematsu N., Iwai H. (1993). Effect of the Addition
of SiO2 and SiO2-CaO on the Reduction of Dense
Wustite at High Temperatures. Tetsu-to-Hagane.

[31] Zheng H., Daghagheleh O., Wolfinger T., Taferner B., Schenk J., Xu R. (2022). Fluidization Behavior and Reduction Kinetics of Pre-Oxidized Magnetite-Based
Iron Ore in a Hydrogen-Induced Fluidized Bed. International Journal of Minerals, Metallurgy and Materials.

[32] El-Geassy A. A., Nasr M. I. (1988). Influence of the Original Structure on the Kinetics
of Hydrogen Reduction of Hematite Compacts. Transactions of the Iron and Steel Institute of Japan.

[33] El-Rahaiby S. K., Rao Y. K. (1979). The Kinetics of
Reduction of Iron Oxides at Moderate
Temperatures. Metall. Trans. B.

[34] Aquilanti V., Coutinho N. D., Carvalho-Silva V. H. (2017). Kinetics
of Low-Temperature Transitions
and a Reaction Rate Theory from Non-Equilibrium Distributions. Philos. Trans. R. Soc., A.

[35] Guo L., Gao J.-t., Zhong S.-p., Bao Q.-p., Guo Z.-c. (2019). In Situ
Observation of Iron Ore Particle Reduction above 1373 K by Confocal
Microscopy. J. Iron Steel Res. Int..

[36] Matsuura H., Tsukihashi F. (2012). Thermodynamic
Calculation of Generation of H 2 Gas
by Reaction between FeO in Steelmaking Slag and Water Vapor. ISIJ International.

[37] Ito K., Fruehan R. J. (1989). Study on the Foaming
of CaO-SiO2-FeO Slags: Part I.
Foaming Parameters and Experimental Results. Metall. Trans. B.

[38] Shi C., Alderman O. L. G., Tamalonis A., Weber R., You J., Benmore C. J. (2020). Redox-Structure Dependence of Molten Iron Oxides. Commun. Mater..

[39] Chen M., Raghunath S., Zhao B. (2013). Viscosity of SiO2-“FeO”-Al2O3
System in Equilibrium with Metallic Fe. Metallurgical
and Materials Transactions B: Process Metallurgy and Materials Processing
Science.

[40] Kirchheim R., Pundt A. (2014). Hydrogen in Metals. Physical Metallurgy: Fifth
Edition.

[41] Luo L., Zhang Z., Li C., Nishu, He F., Zhang X., Cai J. (2021). Insight into Master Plots Method for Kinetic Analysis of Lignocellulosic
Biomass Pyrolysis. Energy.

[42] Zhao, B. ; Jak, E. ; Hayes, P. C. Experimental Investigation of Liquidus Temperatures of ISP Slags. VII International Conference on Molten Slags, Fluxes and Salts; The South African Institute of Mining and Metallurgy, 2004; pp 243–248.

[43] Hong Q. J., Van De Walle A. (2016). A User Guide
for SLUSCHI: Solid and Liquid in Ultra
Small Coexistence with Hovering Interfaces. Calphad.

[44] Heumann, T. ; Mehrer, H. Diffusion in Reinmetallen. Diffusion in Metallen; Springer, 1992; pp 103–130, 10.1007/978-3-642-86413-1_6,

