# Supplementary material for: Bridging the Gap in Carbon Free Iron Making: How Hydrogen Affects the Reduction of Iron Ore between 900 and 1590 °C
Source: ACS Sustain Chem Eng. 2025 Oct 16;13(42):17726–32. doi: 10.1021/acssuschemeng.5c03402 (PMC12570257; doi:10.1021/acssuschemeng.5c03402)
Supplement: Supplementary file 1 [file sc5c03402_si_001.pdf]

Supplementary Information

Bridging the Gap in Carbon Free Iron Making: How Hydrogen Affects the Reduction of Iron Ore between 900 and 1590 °C

Ram Krushna Mohanta,[1] Dallin Fisher,[1] Yuri Korobeinikov,[1] Qijun Hong,[1] Christopher Muhich,[1] Seetharaman Sridhar,[1] Noemi Leick [2] *

[1] *Arizona State University, Tempe, AZ 85281, USA*

[2] *National Renewable Energy Laboratory, Golden, CO 80401, USA.*

* *Corresponding Author Email: noemi.leick@nrel.gov*

The supplementary information file includes:

1. Experimental Methods and Materials……………………………………………………..S3
   1.1. Experimental Methods (**Figure S1**)…………………………………………………S3
   1.2. Computational Method to model O_2 diffusion in BCC and FCC iron…………………S4
   1.3. Measurement of porosity using MATLAB® image analysis technique **(Figure S2)**……..S5
   1.4. Material composition **(Table S1)**……………………………………………………S6
2. **Figure S3**: In-situ images obtained by the CSLM of the iron ore samples reduced for 20 min with 5% H_2 at specific temperatures between 900 and 1590 °C…………….……………S7
3. Additional Information for Figure 2 (a)……………………………..…………….S8
4. **Figure S4:** Reduction degree as a function of temperature after H_2 exposure times varying between 10 min and 120 min, which the 20 min data being the same as that presented in Figure 1. For technical reasons, it is not possible to expose samples to temperatures beyond 1400 °C for durations of 40 min or longer, which is why there are no datapoints for these conditions………………………………………………………..……..…………….S9
5. **Figure S5**: Melting point of slag as a function of FeO wt% in slag as predicted by FactSage 8.2 in 5% H_2 atmosphere……………………………………………………………...S10
6. **Figure S6:** Percentage of metallization after reduction at different time for different temperatures……………………………………………………………………...S11

7. **Figure S7:** Calculated diffusion coefficients of oxygen (with initial oxygen concentration set to x_O=7.2%) in BCC and FCC iron at 1300, 1500, and 1700 K. Four independent DFT MD simulations were performed at each temperature and phase. Oxygen diffusion data are shown in red, while iron diffusion data are in green. Our results indicate significantly higher diffusion coefficients in BCC iron……………………………………………………………….……S12
8. **Figure S8:** In-situ images obtained by the CSLM of the iron ore samples at different time interval for different temperature ranges (900-1590 °C). The images for temperature above 1500 °C are limited to 40min due to reduction experiment not conducted beyond 40 min owing to safety reasons………………………………………………………………………….S13
9. **Reaction Kinetics Analysis**………………………………………………………………..S14
   9.1. **Figure S9**: Different reaction mechanism model as mentioned in Table S2 in integral form……………………………………………………………………………………S16
   9.2. **Figure S10**: Master Plot analysis of H_2 reduction of iron ore at various temperatures. Alpha was defined as the fraction of Fe reduced compared to the total possible Fe reduction.………………………………………………………………………………S16
   9.3. **Figure S11:** Master Plot analysis of H_2 reduction of iron ore at various temperatures. Alpha was defined as the fraction of Fe reduced compared to the total Fe reduction in the experiments.…………………………………………………………………………S17
10. **Figure 12:** Phase diagram shows the low solubility of oxygen in Fe...............................S19
11. **References**…………………………………………………………………………….S20

1. Experimental methods & Materials

1.1. Experimental Method

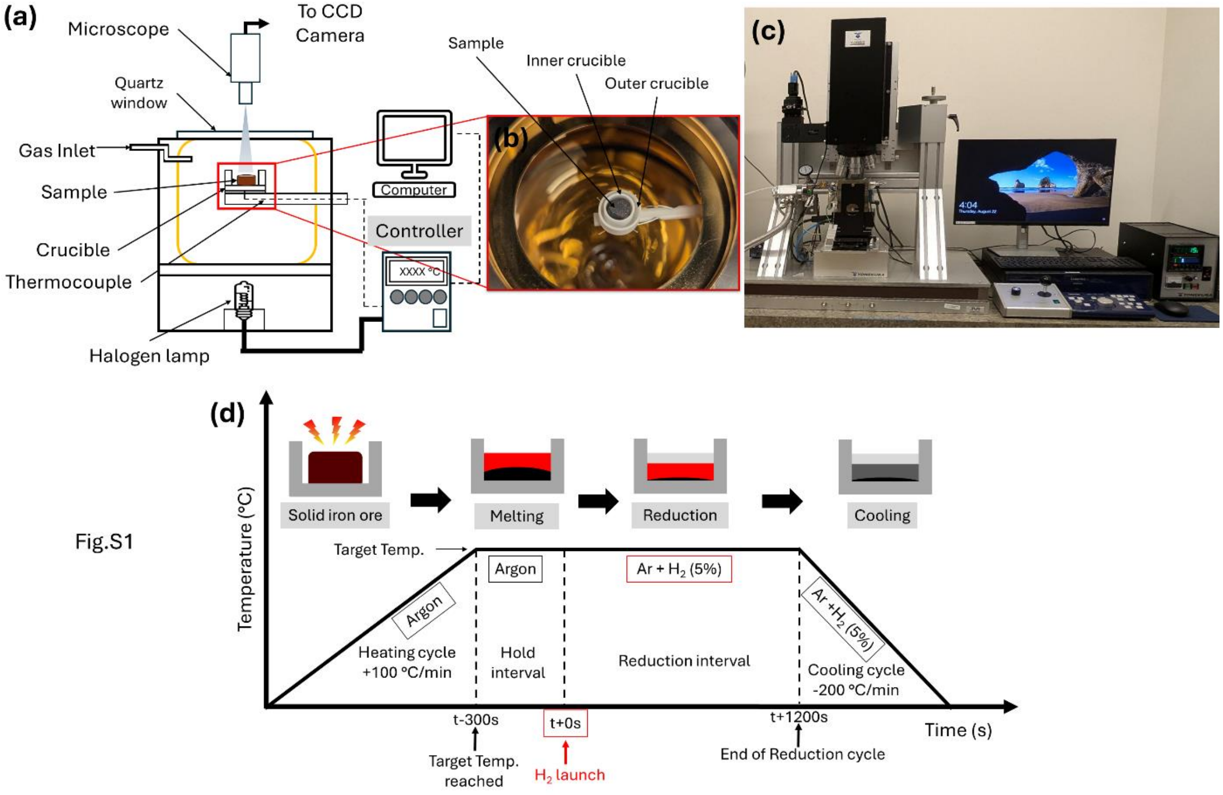

Figure S1: (a) Schematics of the ultra-high temperature confocal scanning laser microscope (CSLM) **(b)** Crucible arrangement for higher temperature experiments **(c)** Photograph of HT-CSLM unit **(d)** Experimental cycle for the high temperature iron ore hydrogen reduction experiment

An Ultra High Temperature Confocal Scanning Laser Microscope (UHT-CSLM)[1] was used for the experiments. The iron ore sample was placed inside an alumina crucible, which was placed on a platinum plate mounted on an alumina cylindrical tube within the heating chamber of the CSLM. An R-type thermocouple was embedded within the alumina tube to monitor the temperature at the base of the crucible, providing feedback for precise temperature measurement control. During the experiments, the reflection from the sample surface was directed through the microscope lens system and focused onto the CCD camera, which was positioned behind a pinhole. **Figure S1(a)** and **(c)** present both a schematic of the UHT-CSLM setup and a photograph of the actual instrument used in this study. The experimental cycle for the high-temperature reduction experiment is presented in **Figure S1(d).** Initially, the iron ore sample was heated at a rate of 100 °C/min until the target temperature was reached. Upon reaching the specified target temperature, the sample was held at that temperature for 300 seconds to achieve thermal equilibrium, termed the "Hold interval." Following this hold interval, the reducing gas mixture, composed of Ar + 5%

H_2 with a constant flow rate of 200 ml/min was introduced at the point labeled as "t+0s." The reduction of the iron ore sample under this gas atmosphere continued for the required period, which corresponds to the "Reduction interval." The reduction process is carried out for time period ranging from 10 – 120 min. The reduction experiments are carried out for different target temperatures between 900 °C and 1590 °C. The experiments above 1500 °C are limited to only 20 min due to the safety of the instrument. At the end of this reduction interval, the sample was rapidly quenched at a cooling rate of 200 °C/min using the same Ar + 5% H_2 gas mixture, to ensure the prevention of any reoxidation during the cooling process. The weight of the sample is measured with the Sartorius weight balance with 0.1mg resolution before and after reduction process for calculation of mass change and reduction kinetics. Equations S1, S2, and S3 define the reduction degree, weight change, and metallization percentage, respectively,

$$\text{Weight change } (\Delta \text{m})\% = \frac{W_{br} - W_{ar}}{W_{br}} \times 100 \qquad \text{Equation S1}$$

$$\text{Reduction degree (RD)}\% = \frac{\Delta \text{m } \%}{29.7} \times 100 \qquad \text{Equation S2}$$

$$\text{Metallization } \% = \frac{Fe_{(total)} - Fe_{(in\ FeO)}}{Fe_{(total)}} \times 100 \qquad \text{Equation S3}$$

where W_{br} and W_{ar}defines the weight of the sample before and after reduction, respectively, $Fe_{(total)}$ denotes the total amount of iron present in the DRI grade hematite ore, i.e. 67.8%, and $Fe_{(in\ FeO)}$ represents the amount of Fe present in oxide form. From the weight change, the fraction of $Fe_{(in\ FeO)}$ and remaining oxygen present in the sample after reduction can be estimated.

After the reduction, the solidified samples were embedded into epoxy mold followed by polishing and metallographically prepared for high-resolution scanning electron microscopy (SEM) analysis and energy dispersive X-ray spectroscopy (EDS).

1.2. Computational Method to model O_2 diffusion in BCC and FCC iron

Here, direct density functional theory (DFT) molecular dynamics (MD) simulations are employed to study the diffusion of oxygen in bcc and fcc iron.. The simulations are performed at 1300, 1500, and 1700 K (1027, 1227, and 1427 °C) with 5 oxygen atoms in 129 and 149 iron atoms in bcc and fcc iron, respectively, which corresponds to an oxygen composition of approximately 3.5%. DFT MD calculations are performed by the Vienna Ab initio Simulation Package (VASP)[2], with the projector-augmented-wave (PAW)[3] implementation and the generalized gradient approximation (GGA) for the exchange-correlation energy, in the form known as Perdew, Burke, and Ernzerhof (PBE)[4]. The electronic temperature is accounted for by

imposing a Fermi distribution of the electrons on the energy level density of states, which is consistent with the ionic temperature in MD. The plane-wave energy cutoff is set to the default value (normal precision) of the pseudo-potential during the MD simulations, and it is further increased to the high-precision value during the correction for the Pulay stress[5]. DFT MD is utilized to simulate atomic movements and trajectories. Specifically, MD simulations are carried out under the constant number of atoms, pressure, and temperature condition (NPT, isothermal and isobaric ensemble). Here the thermostat is conducted under the Nose-Hoover chain formalism[6–8]. The barostat is realized by adjusting volume every 80 steps according to average pressure. Although this does not formally generate an isobaric ensemble, this approach has been shown to provide an effective way to change volume smoothly and to avoid the unphysical large oscillation caused by commonly used barostat[9]. We perform the DFT MD simulations through the SLUSCHI package10, which automates the process and the calculation of diffusion coefficient[10].

1.3 Measurement of porosity using MATLAB® image analysis technique

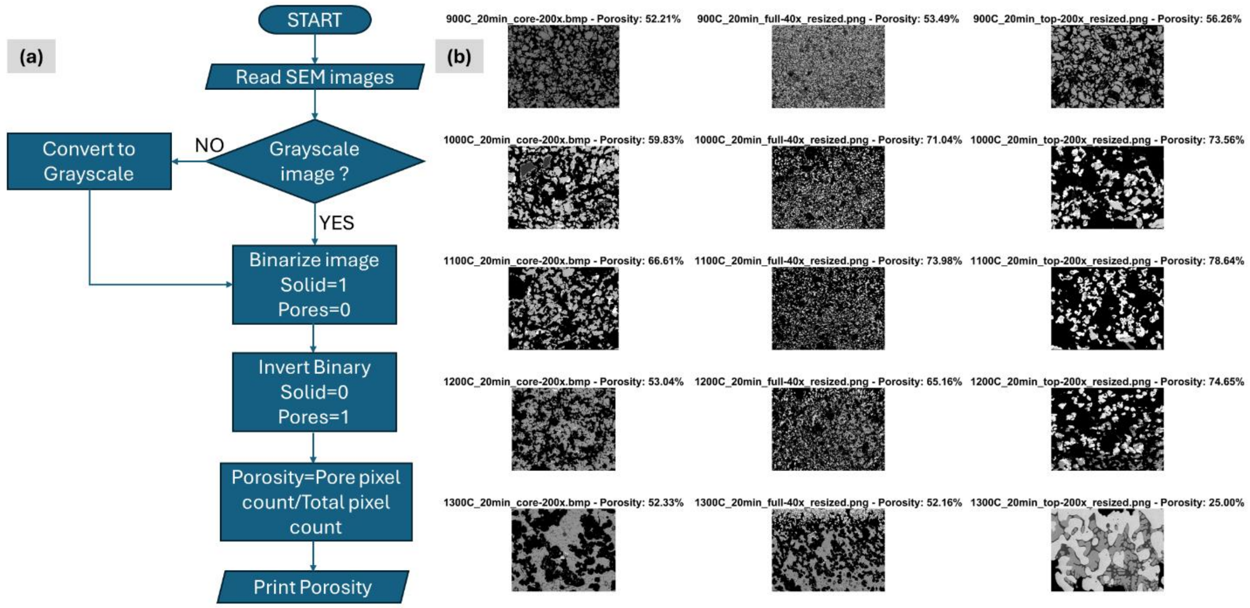

Figure S2: (a) Algorithm structure of the MATLAB image analysis technique for porosity measurement (b) Output of the MATLAB image analysis technique; Core-200X: Core region image at 200X magnification, Full-40X: Full Image at 40X magnification, top-200X: Edge region at 200X magnification

Porosity was estimated using a custom MATLAB-based image analysis algorithm (outlined schematically in Figure below), which processes SEM micrographs through a series of steps: grayscale conversion (if needed), contrast enhancement, adaptive thresholding for binarization, inversion of binary images, and finally porosity computation as the ratio of pore pixel count to total pixel count. This approach, while semi-quantitative, is particularly suited for small and thermally processed specimens where standard porosimetry

methods are impractical. To account for spatial variation, SEM images were collected from three representative regions—core, edge, and full cross-section—at each temperature condition. Porosity was calculated independently for each region and plotted against temperature.

1.4 Material Composition

The Direct Reduced Iron (DRI) grade hematite pellets used in this study were supplied by Voestalpine, USA [11]. The chemical composition of the hematite ore is provided in **Table S1**. They have a typical density of 3.55±0.1 g/cm3, total porosity of 31±2% and open porosity of 28%. The pellets were sectioned into thin cylinders with a diameter and height of < 8 mm each. For target temperatures of 900-1400 °C, the sample weight close to 160 mg, with a maximum sample-to-sample difference of 12 mg. For experiments conducted at higher target temperatures, 1500, 1550, and 1590 °C, where the sample transitions to a primarily liquid state during reduction, an additional alumina crucible (Ø8 mm x 6 mm) was employed to prevent slag leakage and protect the sample holder (Figure S1 (c)). Due to the inclusion of this inner crucible, the sample mass was halved. Ultra-high purity argon gas (99.999%, supplied by Matheson) was used during the initial heating cycle at a 200 ml/min flow rate and for the reduction and cooling cycles, a gas mixture of argon with 5% hydrogen (supplied by Matheson) was used with 200 ml/min.

Table S1 Chemical composition of DRI grade iron ore, wt. %

Fe (total)	SiO_2	CaO	Al_2O_3	MgO	TiO_2	Volatiles
67.8 %	1.34%	0.76%	0.49%	0.01%	0.1%	0.48%

*Maximum amount of oxygen present in Fe_xO_y = 29.7%

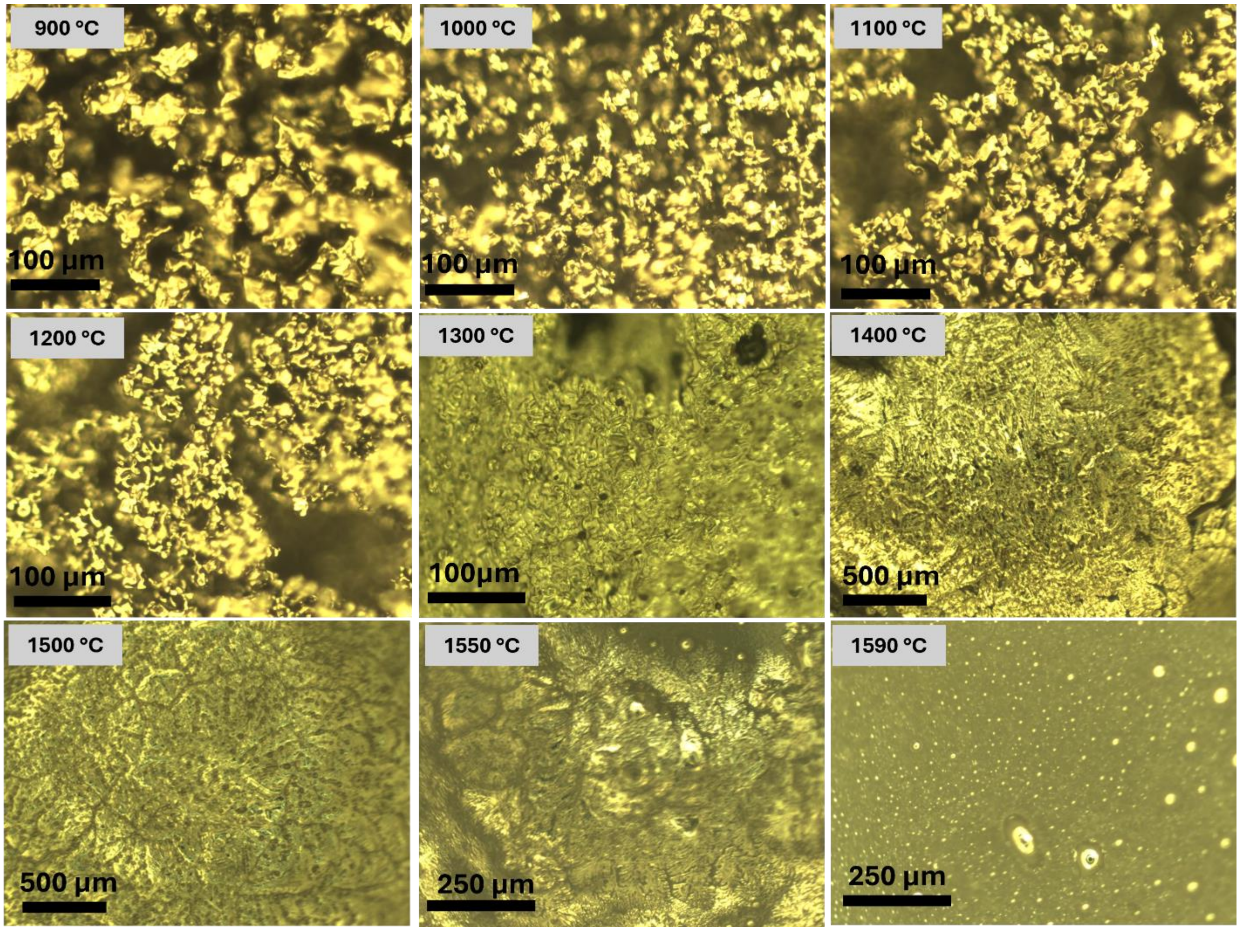

Figure S3: In-situ images obtained by the CSLM of the iron ore samples reduced for 20 min with 5% H_2 at specific temperatures between 900 and 1590 °C.

2. Additional Information for Figure 2 (a):

The equilibrium phases of the iron ore are predicted by the FcatSage 8.2 in an argon environment with oxygen (O_2) partial pressure maintained at 10^{-6} atm from 900 – 1590 °C. The predicted phases show the liquid slag formation starts at 1125 °C and gradually increases until 1350 °C. By 1406 °C, the sample consists of approximately 94% liquid slag, with FeO and Fe_2O_3 comprising 60 wt% and 37 wt%, respectively. As the temperature rises further, the Fe_2O_3 content in the liquid slag decreases, while FeO increases.

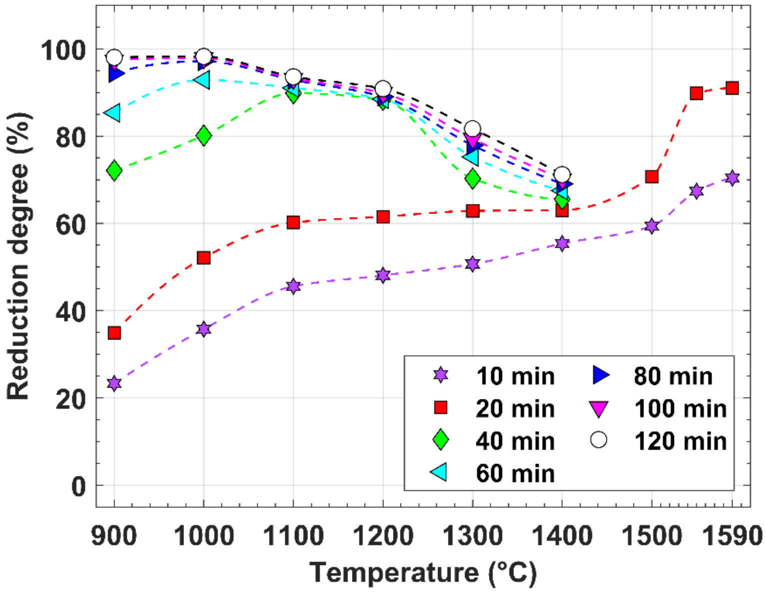

Figure S4: Reduction degree as a function of temperature after H_2 exposure times varying between 10 min and 120 min, which the 20 min data being the same as that presented in Figure 1. For technical reasons, it is not possible to expose samples to temperatures beyond 1400 °C for durations of 40 min or longer, which is why there are no datapoints for these conditions

Reduction experiments above 1400 °C for durations exceeding 40 minutes cannot be performed because, under these conditions, the slag becomes highly reactive due to its high FeO content. This increases the likelihood of a reaction with the alumina crucible, which can lead to cracking and potential leakage of high-temperature molten slag.

However, at temperatures above 1500 °C, the reduction degree reaches approximately 90% within just 20 minutes, indicating that nearly all the iron ore has been reduced, signifying equilibrium conditions. Therefore, the plot in **Figure S3** is extrapolated for such conditions.

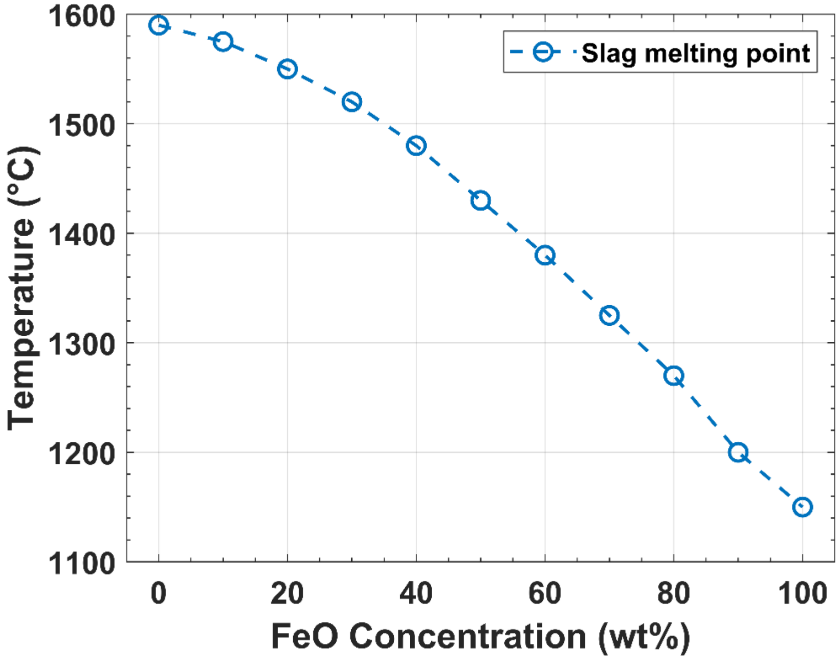

Figure S5:Melting point of slag as a function of FeO wt% in slag as predicted by FactSage 8.2 in 5% H_2 atmosphere.

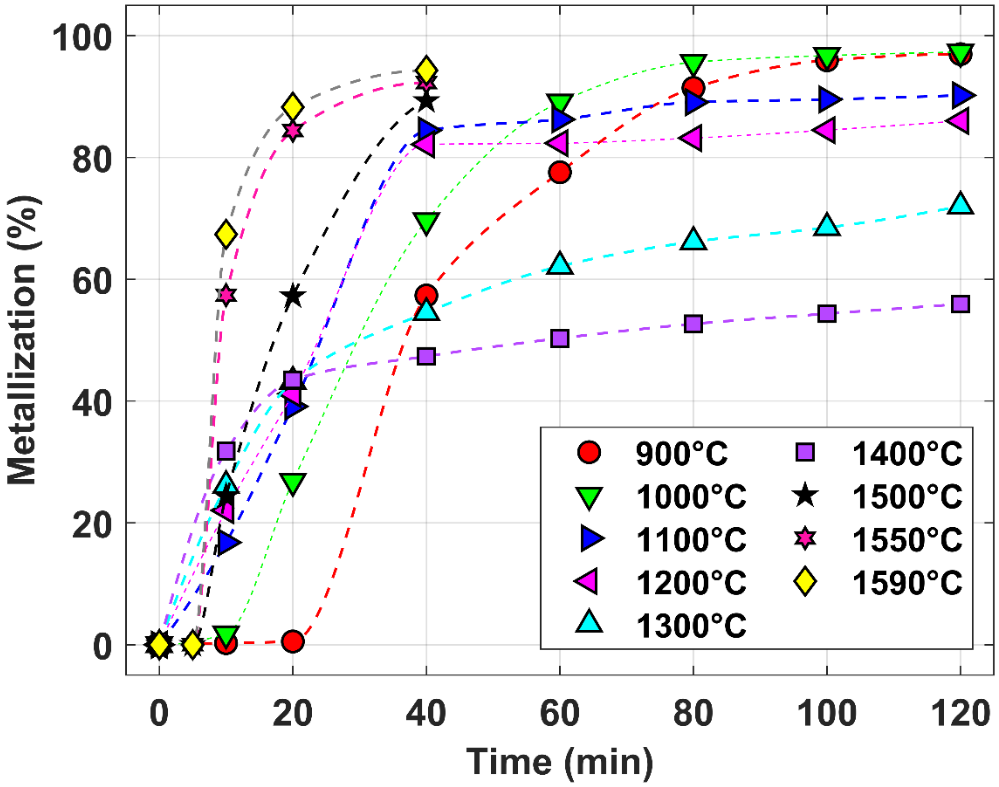

Figure S6: Percentage of metallization after reduction at different time for different temperatures

Figure S6 depicts the metallization degree, representing the percentage of iron reduced to its metallic state after 20 minutes exposure to H_2. The low metallization below 1100 °C suggests that the reduction process is still in its early stages, with much of the sample either unreduced or in intermediate oxide forms. Similar to the RD curve, metallization increases gradually between 1100 °C and 1400 °C. At 1540 °C and above, metallization rises sharply, reaching 86% at 1590 °C, indicating near-complete reduction of iron oxides to metallic iron (Fig. 1(a)). This steep increase in metallization is attributed to the accelerated reduction kinetics at high temperatures, where iron oxides approach their melting points, facilitating rapid oxide-to-metal conversion and easy phase separation.

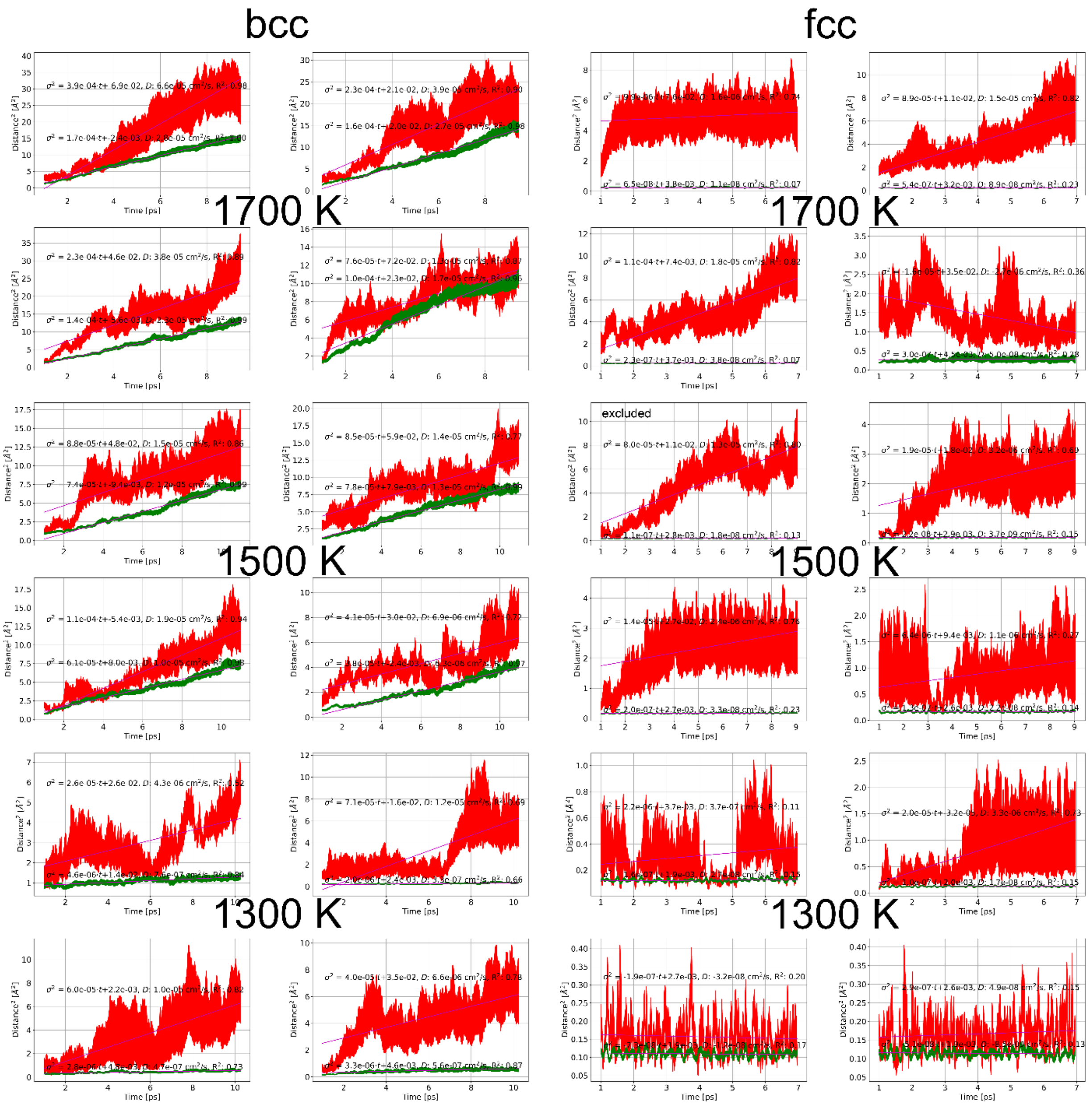

Figure S7: Calculated diffusion coefficients of oxygen (with initial oxygen concentration set to x_O=7.2%) in BCC and FCC iron at 1300, 1500, and 1700 K. Four independent DFT MD simulations were performed at each temperature and phase. Oxygen diffusion data are shown in red, while iron diffusion data are in green. Our results indicate significantly higher diffusion coefficients in BCC iron.

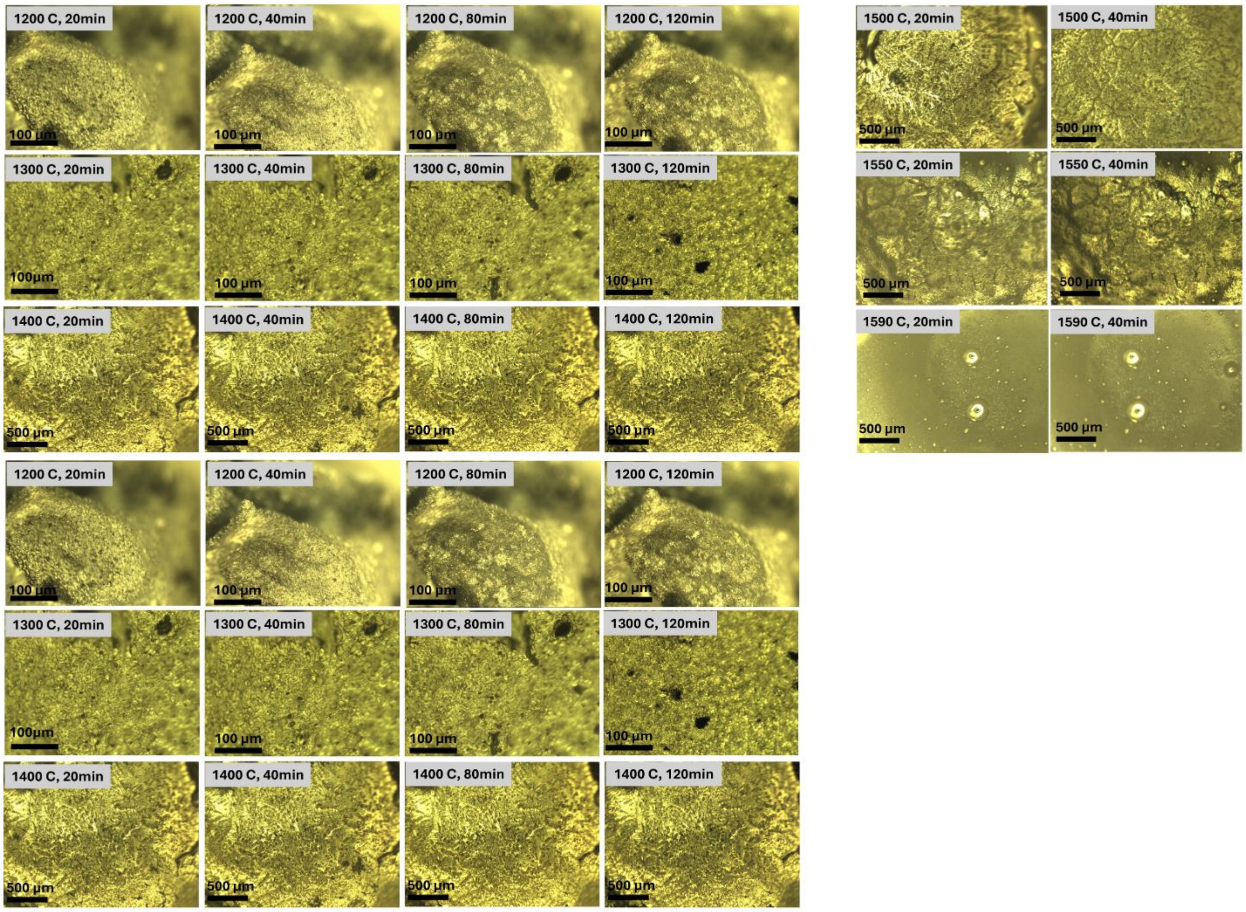

Figure S8: In-situ images obtained by the CSLM of the iron ore samples at different time interval for different temperature ranges (900-1590 °C). The images for temperature above 1500 °C are limited to 40 min due to reduction experiment not conducted beyond 40 min owing to safety reasons.

3. Reaction Kinetic Analysis:

A detailed analysis of the reduction extent with time was conducted in an attempt to extract the kinetics controlling H_2 reduction of the complex iron ore. Specifically, master plot analysis was used, focusing on the integrated formalism, to extract the reaction mechanism. Master plot analysis assumes a functional form for the reaction of:

$$\frac{d\alpha}{dt} = k\,f(\alpha) \qquad \text{Equation S4}$$

where k is the reaction rate:

$$k = A\exp\left(-\frac{E_a}{RT}\right) \qquad \text{Equation S5}$$

and $f(\alpha)$ describes the kinetic mechanisms, i.e. how the rate changes with reaction extent (α). A set of common mechanisms was examined which generally control gas/solid particles reactions. Generally, these models can be classified by the governing mechanisms: i.e. surface reactions, auto-catalytic reactions, nucleation controlled, reaction across a contracting interface (e.g. shrinking core), and diffusion. Within each class, there are different orders. These are outlined in Table S2.

Table S2: Reaction mechanisms examined in Master Plot analysis

**Type**	**Name**	**Abbreviation**	**f(α)**
Surface reaction	First-order	F1	$(1-\alpha)$
	Second order	F2	$(1-\alpha)^2$
	Third order	F3	$(1-\alpha)^3$
Autocatalytic	Autocatalytic	B1	$\alpha(1-\alpha)$
Nucleation	Avrami Erofeev	A1.5	$1.5\,(1-\alpha) * [-\ln(1-\alpha)]^{1/3}$
	Avrami Erofeev	A2	$2\,(1-\alpha) * [-\ln(1-\alpha)]^{1/2}$
	Avrami Erofeev	A3	$3\,(1-\alpha) * [-\ln(1-\alpha)]^{2/3}$
	Avrami Erofeev	A4	$4\,(1-\alpha) * [-\ln(1-\alpha)]^{3/4}$
Contracting Geometry	Contracting plane	R2	$2\,(1-\alpha)^{1/2}$

	Contracting surface	R3	$3\ (1-\alpha)^{2/3}$
Diffusional	1D diffusion	D1	$\frac{1}{2\alpha}$
	2D diffusion	D2	$\frac{1}{[-\ln(1-\alpha)]}$
	3D diffusion	D3	$\frac{3(1-\alpha)^{2/3}}{2\ [1-(1-\alpha)^{1/3}]}$
Power law	2/3s Power	P 2/3	$2/3\ (\alpha^{1/2})$
	2nd order Power	P2	$2\ (\alpha^{1/2})$

By normalizing the reaction rate with the expected rate at $\alpha = 0.5$, the rate constant cancels out of the equation and the resulting ratios are only mechanism dependent. Thus, the mechanism can be easily deduced by plotting this ratio against α.

$$\frac{\frac{d\alpha}{dt}(\alpha)}{\frac{d\alpha}{dt}(\alpha = 0.5)} = \frac{f(\alpha)}{f(\alpha = 0.5)} \qquad \text{Equation S6}$$

This formulation emphasizes differences between models at $\alpha < 0.5$. However, determining $\frac{d\alpha}{dt}(\alpha)$ from experimental data requires taking a numeric derivative, which can induces substantial error, especially over widely spaced time intervals. Further, $\alpha = 0.5$ is often not directly available from experiment, and thus is found by interpolation, from which numeric derivatives must be taken.

A similar analysis and construction can be conducted to generate an integral form.

$$g(\alpha) = \int_0^\alpha \frac{d\alpha}{dt} = A * \theta \qquad \text{Equation S7}$$

As with the differential form, the integral form can be normalized by division against an specified reaction extent. However, unlike the differential form, only the interpolation to $\alpha = 0.5$ is required. As such, we adopted the integral form for our analysis. This formulation emphasizes behavior at $\alpha > 0.5$

$$\frac{g(\alpha)}{g(\alpha = 0.5)} = \frac{\theta}{\theta(\alpha = 0.5)} \qquad \text{Equation S8}$$

A plot of the different models in integral form is shown in Figure S9.

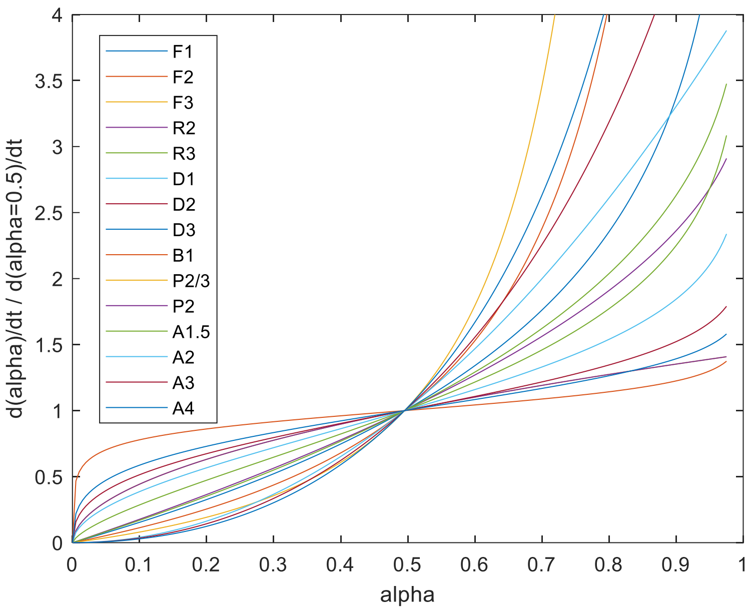

Figure S9: Different reaction mechanism model as mentioned in Table S2 in integral form

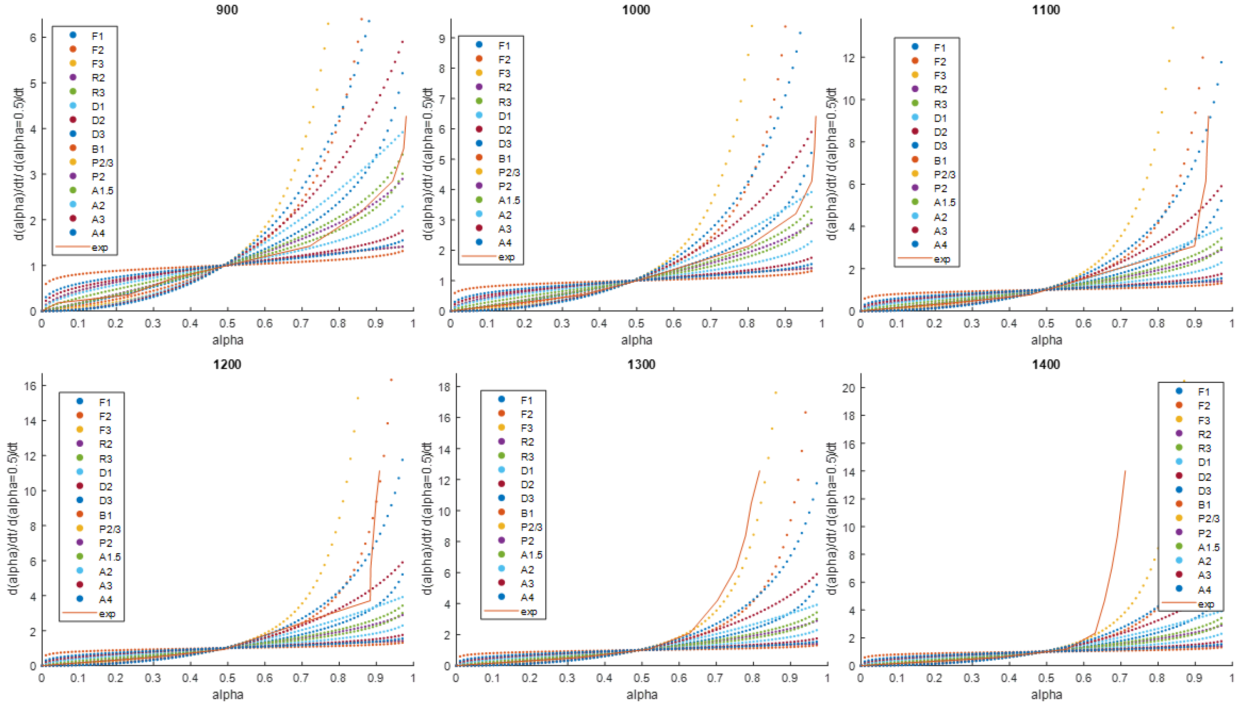

Figure S10: Master Plot analysis of H_2 reduction of iron ore at various temperatures. Alpha was defined as the fraction of Fe reduced compared to the total possible Fe reduction.

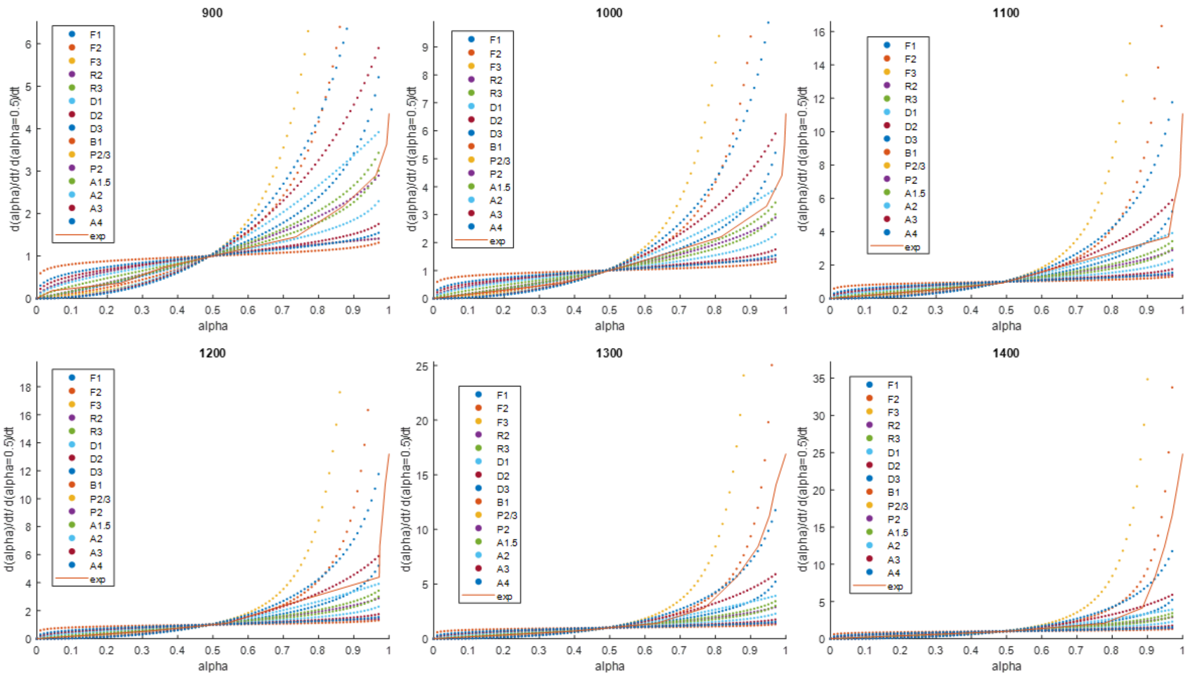

Figure S11: Master Plot analysis of H_2 reduction of iron ore at various temperatures. Alpha was defined as the fraction of Fe reduced compared to the total Fe reduction in the experiments.

To determine the active mechanism, we plotted the experimental $g(\alpha)$ on the same master plots for the various temperatures. Lagrangian interpolation was used to find $\alpha = 0.5$. Two approaches were taken. Firstly, referencing alpha to complete reduction based on the weight, as shown in Figure S10, and secondly, the more traditional approach of normalizing the reaction to the maximum reaction extent achieved, Figure S11. The latter accounts for the fact that at high temperatures the reaction stopped well short of complete reduction, likely due to the formation of a passivating skin.

As can be seen, the behavior is quite complex, and inconsistent both between temperatures and even at a given temperature. For example, at 1000 °C, the reaction most resembles an A1.5 mechanism early but then transitions to more D3 behavior at the end. The reaction behavior appears to be more diffusion, nucleation, or shrinking interface at lower temperatures, but transitions to more surface limited reactions at higher temperatures.

The difficulty in assessing the behavior likely arises for two-fold reasons. The underlying material itself is quite complex given its mixed composition, and its reduction requires multiple transport, surface, and nucleation processes which at any time could change the governing mechanism. Secondly, the temperatures over which this analysis was completed go through different melting temperatures. The formation of multiple phases, some of which are liquid and some are solid, at various points along the reaction obfuscates the analysis. Further, because the reaction mechanisms are certainly different between each of the

temperatures examined, it is unreasonable to attempt to extract an activation barrier or pre-exponential factor.

These findings suggest that more detailed data collection is required if kinetics are to be extracted. Firstly, new data sets must be developed at finer time spacings. Such data set would both improve the $\alpha = 0.5$ estimate and provide more points for matching. This would also enable finer analysis of where controlling behavior transitions. Further, the derivative form could be used and cross compared. Secondly, much narrower temperature ranges should be examined. In a smaller temperature range, the chances that the reaction mechanism changes drastically between temperature points is lowered, allowing the extraction of reaction barriers and pre-exponential to be extracted. This analysis is left to future endeavors dedicated solely to kinetic extraction.

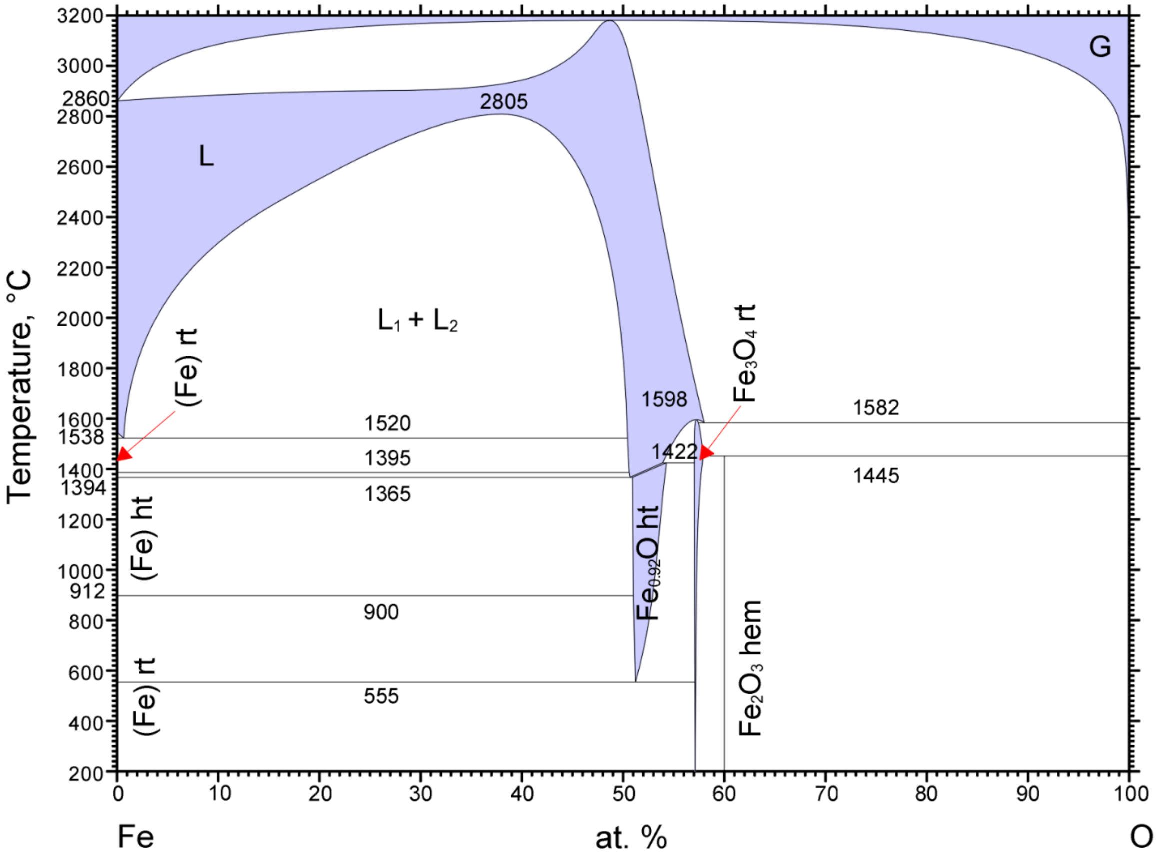

Figure 12: Phase diagram shows the low solubility of oxygen in Fe.

Adding the low solubility in Fe shown in Fig. S12 to Fig. 2b, Fig. S3 and Table 1, a high-level map of sample behavior across temperatures can be provided: 900–1200 °C—high surface area with little to no contiguous Fe barrier; small Fe-layer fractions emerge by 1100–1200 °C and begin to depress the reduction degree. 1300–1400 °C—a continuous γ-Fe (FCC) product layer forms and strongly limits further reduction. 1500 °C—a δ-Fe (BCC) product layer prevails; aided by higher oxygen mobility in BCC, the sample reaches near-complete reduction in ~40 min. 1550–1590 °C—the system enters the liquid regime, where oxygen solubility and transport are much higher, and the overall reaction rate correspondingly accelerates.

References

1 yonekura mfg, ht-cslm, https://yonekuramfg.wixsite.com/ht-cslm, (accessed 20 February 2025).

2 G. Kresse and D. Joubert, *From ultrasoft pseudopotentials to the projector augmented-wave method*, .

3 P. E. Blöchl, *Phys Rev B*, 1994, **50**, 17953–17979.

4 J. P. Perdew, K. Burke and M. Ernzerhof, *Phys Rev Lett*, 1996, **77**, 3865–3868.

5 G. P. Francis and M. C. Payne, *Journal of Physics: Condensed Matter*, 1990, **2**, 4395.

6 S. Nosé, *Mol Phys*, 1984, **52**, 255–268.

7 S. Nosé, *J Chem Phys*, 1984, **81**, 511–519.

8 W. G. Hoover, *Phys Rev A (Coll Park)*, 1985, **31**, 1695–1697.

9 Q. J. Hong and A. Van De Walle, *Journal of Chemical Physics*, 2013, **139**, 94114.

10 Q. J. Hong and A. Van De Walle, *Calphad*, 2016, **52**, 88–97.

11 Nucor | Home, https://nucor.com/, (accessed 22 August 2024).
